# Testing the effectiveness of a health intervention that manipulates the social environment at active leisure events in Scotland

**DOI:** 10.1177/22799036251396734

**Published:** 2025-12-18

**Authors:** Andre Simon Gilburn

**Affiliations:** 1 Biological and Environmental Sciences, Faculty of Natural Sciences, University of Stirling, Scotland, UK

**Keywords:** parkrun, physical activity, active leisure event, mass participation event, social intervention

## Abstract

**Background:**

Active leisure events are health interventions promoting physical activity amongst non-traditional sporting participants. Models of physical activity assume that individual and environmental components interact to shape activity levels. The active leisure event organiser, parkrun, recently introduced a new volunteer role, the parkwalker, to manipulate the social environment at their events to encourage more walkers to attend.

**Design and methods:**

This study compares the finishing times of new parkrun participants in Scotland for a year before and after the introduction of the parkwalker initiative. Different parkrun venues in Scotland were separated into those that fully adopted, partially adopted and did not adopt the parkwalker role into their events.

**Results:**

A model of finishing times revealed they have slowed after the introduction of parkwalkers and the level of slowing is associated with the level of adoption of the role by events. The parkwalker initiative was particularly associated with the slowing of finishing times of older new participants and participants at larger events. The initiative was also associated with an increase in the proportion of female new participants and a reverse in the recent decline in the age of new participants.

**Conclusions:**

The findings suggest that parkrun have introduced a successful intervention to their events that has manipulated the social environment to increase both engagement and inclusivity. This has management implications for both parkrun and other active leisure events. Practitioners engaging in social prescription might want to direct patients towards those active leisure events most engaged in welcoming slower new participants.

**Contribution to Public Health:**

This study investigates how an intervention, the parkwalker Initiative, impacted the demographics of participants at parkrun. The intervention manipulated the social environment at events through using a volunteer walker who encourages other walkers.

This study showed that finishing times slowed at parkrun events after the introduction of the initiative suggesting that a barrier to participation for some walkers had been overcome. The level of engagement with the initiative was associated with the level of slowing in finishing times.

Active leisure events can successfully manipulate their social environment to increase participation by specific target demographics. It was also notable that the slowing involved increases in the proportion of slower runners as well as walkers. The findings have implications for event organisers and practitioners engaging in social prescription of parkrun who might be best directing patients to events with a regular parkwalker.

## Background

Physical inactivity is a key contributory factor in non-communicable disease and mortality.^1–3^ Mortality rates during the COVID-19 pandemic were associated with sedentary behaviour, revealing that increasing physical activity (PA, see Table 1 for a list of abbreviations) would make populations more resilient to future pandemics.^4^ Consequently, increasing levels of PA is an urgent priority to reduce avoidable burdens being placed upon healthcare systems^5^ and reduce mortality rates.^6–8^ An understanding of the factors affecting levels of PA is required to develop and test the effectiveness of health interventions aimed at increasing PA. Models of PA, such as the socio-ecological model, assume that levels of exercise are shaped by individual and environmental factors and interactions between these factors.^9–11^ Environmental factors can include components of the social environment and physical environment.^12–15^

**Table 1.** A list of abbreviations used in the article.

Term	Abbreviation
Active leisure event	ALE
General linear mixed model	GLMM
Physical activity	PA
Parkwalker initiative	PI

Individual factors include gender and age with studies finding men more likely to partake in PA than women and lower levels of PA in older adults.^11,16,17^ The context in which exercise occurs can also vary between the genders, as can the intensity of PA.^18^ Ethnicity is also an important individual factor influencing levels of PA^13,18,19^ with ethnic minorities often displaying lower levels of PA. Social cognitive theory emphasises the importance of self-efficacy as a key factor in determining levels of activity.^12,14,17,20^ Lower levels of PA are also associated with socio-economic status, with lower levels of activity associated with higher levels of deprivation.^16,18,19,21^

The social environment can promote PA where peers encourage PA as part of a group and where feeling a sense of belonging to a community encourages engagement with group events.^13,15,17,20^ The physical environment can include factors such as the proximity and quality of places where people can exercise.^20^ These can be both indoors, such as gyms,^19^ and outdoors, such as urban parks.^22^ For parks and other outdoor areas the amount of green and blue space within them can promote PA.^22^

A key component of theoretical models of engagement with physical activity is that there are considerable interactions between individual and environmental factors and that these play a key role in shaping behaviour.^5,11^ Previous studies have largely focussed on the impacts of altering components of the physical environment and assessing their impacts on PA. For example, some studies have identified that making improvements to public parks resulted in increases in PA in the improved areas of the parks.^23,24^ Other studies have looked at the introduction of entirely new programs designed to increase walking which have had mixed outcomes.^25,26^ There is also evidence that once an intervention ceases the gains in PA are soon lost.^27^ A meta-analysis found that new social walking programs did increase PA in deprived areas but only for adults.^28^ Whilst existing studies have focussed on the impact of the introduction of new initiatives one key knowledge gap is whether long-term health promotion programs can be manipulated to be more effective.

Active leisure events (ALEs) are long term health interventions designed to promote PA. ALEs promote participation in outdoor group exercise among non-traditional sporting participants. By contrast, most mass participation sporting events focus upon promoting competition between participants.^29^ Another advantage of ALEs is that they occur regularly rather than annually like many mass participant events meaning that engagement can become habitual and there is no drop in PA associated with a program ending.

The largest organiser of ALEs are parkrun who host around 2000 weekly 5 km events globally making it an ideal model system for studying how ALEs can function to promote PA.^30,31^ The results of all parkrun events are published which means that assessments about levels of engagement can be derived without any requirement for self-reporting, removing concerns about common method bias that can impact survey based studies.^32^

Despite the name, there is no requirement to run at parkrun events, and walking is actively encouraged.^31^ The participants at parkrun events have been getting progressively slower over time as parkrun has become more inclusive.^33^ There has also been a shift towards parity in the gender ratio from more male dominated events.^33^ Previous studies of parkrun have identified a range of individual and environmental factors that are associated with different measures of participation and which mirror those identified in wider studies of PA suggesting that findings from studies on parkrun are likely to be transferrable. Individuals factors affecting levels of participation in parkrun include age,^34–37^ gender,^34–37^ education level,^38^ socio-economic status,^35,38–41^ self-efficacy,^38^ ethnicity^40^ and level of fitness.^34,37,42^

Many studies have identified perceived social benefits from participating and volunteering at parkrun^38,43–46^ with variation in social identities influencing levels of participation in parkrun and vice versa.^47–49^ Specific social factors have been parameterised in a model of returning to parkrun for new participants, identifying that larger events have lower return rates but events with a higher proportion of other new participants have higher return rates.^34^ Those parkrun events whose routes go through relatively wooded areas and past more freshwater have higher return rates.^34^ For events with high levels of freshwater along the route, the return duration for repeat participation is shorter showing these environmental features are associated with higher levels of continuing engagement.^50^ Another study has shown that the impacts of freshwater and woodland interact during summer months potentially as a result of water bodies reflecting the trees to enhance their impact.^51^ A model of parkrun growth rates in Australia found that blue spaces and tarmac surfaces were associated with increases in attendance.^35^

Very few studies of engagement with parkrun have included interactions so there is a large knowledge gap. The exception is a series of studies of a range of measures of engagement with parkrun in Scotland. These studies have identified interactions between several variables showing that different demographics vary in their response to specific environmental factors.^33,34,50,51^ Identifying such interactions can be key when designing interventions as it is the response of the key target demographics that are important rather any overall trend. Significant interactions identified in these studies include associations between: age and elevation gain which suggests that hills are disproportionately larger barrier to older runners^33^; gender and time with females showing faster increases in fitness than males^33^; gender and the proportion of freshwater on a parkrun route suggesting that the presence of freshwater has a greater impact on the likelihood of returning by female than male first time participants^34^; fitness level and the attendance at an event suggesting that less fit first time participations return more quickly after attending larger events^50^; fitness level and the proportion of woodland and freshwater on a route suggesting that environmental features were more likely to promote a faster return time for fitter first time participants.^50^

The mission of parkrun is to make the world a healthier and happier place and successful health interventions have already been introduced by parkrun. The parkrun practice initiative was jointly established by parkrun and the Royal College of General Practitioners in the UK.^31^ This involved social prescribing parkrun to those with health issues that would be improved by an increase in PA. To enhance the effectiveness of the parkrun practice the demographic characteristics of parkrun participants were identified which revealed that 45% of participants who walk at parkrun have a long-term health condition.^31^ To increase engagement with parkrun by people with long-term health conditions in England the PROVE project was launched involving the use of outreach ambassadors with specific knowledge of a range of different long-term health conditions to identify ways of making parkrun more inclusive to these key demographics.^52^

In October 2022 parkrun introduced the parkwalker initiative (PI).^53^ The aim of this initiative was to increase the number of participants at parkrun who walk by creating a new volunteer role, the parkwalker. The parkwalker role involves a volunteer walking at the event wearing a distinctive vest to identify themselves as a parkwalker. The parkwalkers are asked to engage in conversation with other participants walking at the event to provide encouragement and support. The PI is an intervention designed to manipulate the social environment at parkrun events to increase inclusivity through making walkers feel more welcome and an important part of the parkrun community.

General Linear Mixed Models (GLMMs) of PA allow parameterisation of the factors shaping levels of PA meaning the relative impacts of individual factors and their interactions can be quantified.^34,50^ The primary aim of this study is to determine the impact of the PI by including it in a GLMM of the finishing time of new participants to parkrun to determine if it has successfully attracted more slower runners and walkers to parkrun. The study will build upon previous GLMMs of parkrun participation and include several individual factors together with components of the physical and social environment that have previously been identified as being associated with levels of participation.^34,50^ The study will also determine if there are interactions between these various factors. The outcomes will determine if the organiser of an ALE can successfully manipulate the social environment at their events to increase engagement by specific target demographics. It will also parameterise any impacts to allow comparisons of the strengths of associations of various factors with the return rate of new participants to parkrun, to inform ALE organisers of how best to optimise the operation of their events to promote engagement by those target demographics.

## Methods

### Data sources

The study included data from all sixty-eight 5km parkrun course venues that held a parkrun event in Scotland over the 2-year period from October 2021 to September 2023. The event results pages were accessed and processed using an Excel macro which extracted information about each participant including their age category, parkrun ID number, gender, age group, finishing time, date and whether the participant was a new parkrunner. The data collection was approved by the University of Stirling ethical review process project number EC 2022 10861 8035. Any unknown participants (participants who completed the event without presenting an identification number) and participants under 18 years of age were removed from the dataset. Those participating in their first parkrun were identified amongst the remaining participants. The dataset consisted of 31,740 adult participants made up of 16,805 females and 14,935 males. These were drawn from 6275 individual events from 68 parkrun venues.

A complete list of the variables used is provided in Table 2. Age for adult participants is provided in the parkrun results as a 5-year cohort except for 18–19 year olds where it is provided as a 2-year cohort. Age was converted to a continuous variable by assigning participants the mid-point age for their cohort.

**Table 2.** The list of predictors included in the general linear mixed models of finishing time, gender and age of new participants to parkrun.

Predictor	Type	Effect	Description
Event	Factor	Random	Name of the event
Date	Numeric	Fixed	Days from start date of study
Individual factors			
Age	Numeric	Fixed	Mid-point of age category range
Gender	Factor	Fixed	Gender as selected by participant at registration
Physical environmental factors			
Elevation gain	Numeric	Fixed	Metres of elevation gained on route
Remoteness (Travelling time to next nearest parkrun)	Numeric	Fixed	In minutes assuming no delays
Surface type	Factor	Fixed	Hard, mixed or soft
Habitat variables	Numeric	Fixed	Proportion woodland and freshwater
Social environmental factors			
Number of participants	Numeric	Fixed	Size of the field
Proportion of new adult participants	Numeric	Fixed	Proportion of field made up of new adult first time participants (%)
Before or after introduction of PI	Factor	Fixed	
Level of adoption of PI	Numeric	Fixed	On roster, on roster but optional, not on roster
SIMD	Numeric	Fixed	Scottish Indicator of Multiple Deprivation Index of area in which parkrun is located

Additional characteristics were collected for the first event each of the participants attended. These were: the number of participants; the number of new adult participants; elevation gain in m; surface type derived from the course descriptions available at parkrun.org.uk and scored 0 for soft surfaces such as trail, 1 for mixed soft and hard surfaces and 2 for hard surfaces such as tarmac; the minimum travelling time in minutes by car from the recommended parking of an event to the recommended parking of another parkrun events determined from Google Maps. This was used as a measure of the remoteness of an event from other parkruns to determine if the parkwalker initiative is equally effective in isolated communities and city centre locations.^33^ Two variables represented the new PI. The first was a categorical variable demarking whether an event took place before or after the introduction of the PI. The second was a score of the level of adoption of the PI by each event. Each event has a published roster which lists the volunteer roles used by that event. These are categorised as either required or optional on the roster page. A score of 2 was given to event venues where the parkwalker was being considered a required role. A score of 1 was given to event venues where the parkwalker was listed on the event roster as an optional role and a score of 0 for events where the parkwalker role was not listed on the roster. The census period for the roster data was the last 6 weeks of the study period as this was the only data publicly available at the end of the study.

### Statistical methodology

The data were analysed using R x64 4.1.1.^54^ Generalised linear mixed models (GLMM) were used to model the number of attendees and the number of new participants to parkrun at each event. GLMMs were also used to model the finishing time of new participants, the gender of new participants and the age of new participants. These models were generated using the lmer and glmer functions in the lme4 package.^55^ As multiple event venues were used, and variation between them was not of primary interest, event venue was included as a random effect in all models to take into account variation between event venues. The model of gender used a binomial error distribution, the model of the number of new participants a poisson error distribution and all other models a Gaussian error distribution. All continuous explanatory variables were scaled to have a mean of zero and a standard deviation of 1.^56^ This generates model coefficients that are directly comparable and makes interpretation easier. A backwards elimination model selection process was adopted with the least significant term being removed until only significant terms remained.^57^ Pairwise correlations were calculated prior to the inclusion of fixed factors to avoid the inclusion of pairs of continuous variables that were correlated beyond a threshold of 0.25. A full list of the individual and environmental predictors included in the starting models is provided in Table 2. Several interaction terms were included in the starting models. These were all possible two-way interactions with the two measures of PI and all possible two-way interactions with age and gender to determine if different demographics respond differently to PI and other factors. Although the models contain more terms and interactions than standard interrupted time series analyses they do contain the key elements of an interrupted time series of comparing the level and slope of the relationship of interest with time both before and after the introduction of the PI.

## Results

### Has the PI impacted attendance figures and the number of new participants?

A GLMM of attendance retained the PI but did not retain date. An interrupted time series model when the PI was introduced produced a better fit to the data than a model with a steady increase over time (estimate = 0.084, s.e. = 0.002, *p* < 0.001). The distance to the nearest parkrun was negatively associated with attendance, with remote events having smaller fields (estimate = −0.033, s.e. = 0.010, *p* = 0.001). The level of adoption of the PI was also positively associated with an increase in attendance (estimate = −0.204, s.e. = 0.097, *p* = 0.034).

A GLMM of the number of new participants at events found an interrupted time series at the point the PI was introduced produced a better fit than a steady increase over time (estimate = 0.076, s.e. = 0.012, *p* < 0.001). Events with larger attendances had more new participants (estimate = −0.451, s.e. = 0.008, *p* < 0.001). A non-significant association with travelling time to the next nearest parkrun was also retained in the model (estimate = −0.010, s.e. = 0.061 *p* = 0.096).

### The finishing time of new parkrun participants in Scotland

The proportion of new parkrun participants who completed their first parkrun event in under 30 min reduced from 46.7% to 42.5% in the year after the introduction of the PI showing that the proportion of faster new participants dropped substantially. The proportion of slower runners who finished between 30 and 50 min increased om 48.0%–49.9%. The proportion of walkers (those who finished after 50 min) increased significantly from 5.2% to 7.6% (683 of 13,056 to 1414 of 19,684). The proportion of walkers increased by 22% at events that did not engage with the PI showing that the proportion of walkers was increasing organically anyway. However, it increased by 54.6% and 55.3% respectively at events that partially and fully engaged with the PI.

A model of the finishing time of new adult parkrun participants in Scotland before and after the introduction of the PI revealed several individual, social and physical factors were maintained in the model (Table 3). The model also retained several interaction terms between factors. As the factors were all normalised the estimates not only determine the direction of any associations but also their relative strength.

**Table 3.** A GLMM of the finishing time of adult first-time participants to parkrun in Scotland. All continuous explanatory variables were scaled. Covariates listed in Table 2 but not present in this table were not significantly associated with finishing and were excluded from the final model.

Parameter	*Z*	Estimate	Standard error	*p*
Intercept	141.46	36.400	0.257	<0.001
Gender (male)	78.38	−6.717	0.086	<0.001
Remoteness	8.06	2.931	0.364	<0.001
Age	32.10	2.597	0.081	<0.001
Proportion of first-timers	12.13	0.670	0.055	<0.001
Parkwalk	5.16	0.659	0.128	<0.001
Number of participants	4.76	0.623	0.131	<0.001
Level of adoption of parkwalk	3.66	0.450	0.123	<0.001
Number of participants × proportion of first-timers	10.19	0.531	0.052	<0.001
Number of participants × remoteness	7.84	2.166	0.028	<0.001
Age × remoteness	5.28	0.203	0.038	<0.001
Age × parkwalk	4.74	0.418	0.089	<0.001
Gender (male) × Age	4.20	−0.364	0.087	<0.001
Age × number of participants	4.13	0.189	0.046	<0.001
Number of participants × level of adoption of parkwalk	3.60	0.275	0.076	<0.001
Gender (male) × number of participants	3.16	0.275	0.087	0.002

**Figure 1.**
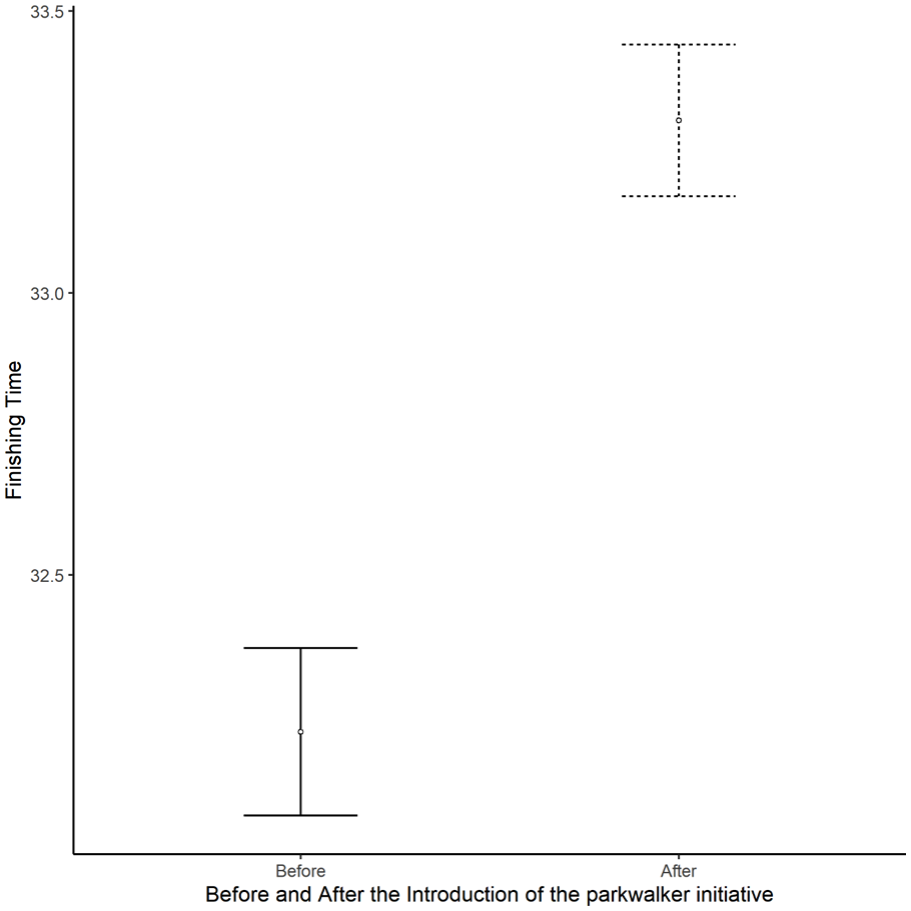
The mean finishing time of new adult parkrun participants before (solid) and after (dashed) the introduction of the PI. Error bars display standard errors.

**Figure 2.**
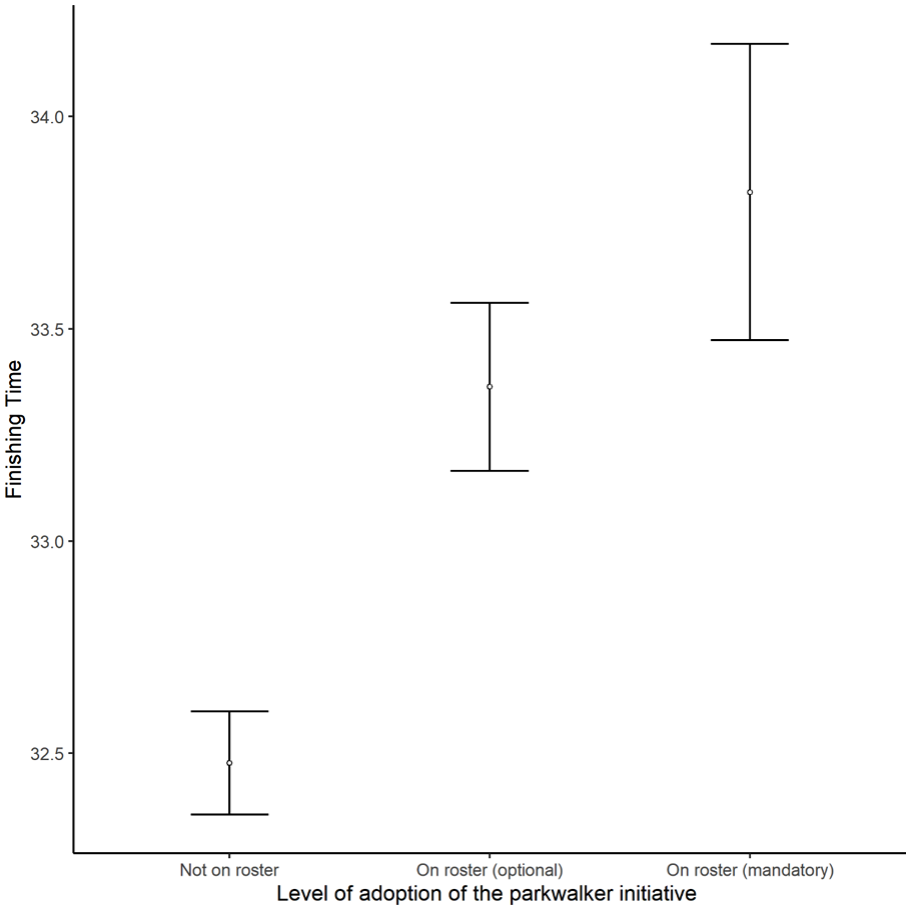
The mean finishing time of new adult parkrun participants after the introduction of the PI at events that adopted the initiative at different levels. Error bars display standard errors.

The most important factor was gender with new female participants taking longer to finish (Table 3). More remote events were found to have significantly slower finishing times. Older new participants took longer to finish. Slower times were recorded by new participants at events where there were a higher proportion of new adult participants. The introduction of the PI was highly significantly associated with slower finishing times (Figure 1). Larger events were also associated with slower finishing times. The final factor retained in the model was the level of adoption of the PI with a highly significant association between the level of adoption and slow finishing times (Figure 2).

**Figure 3.**
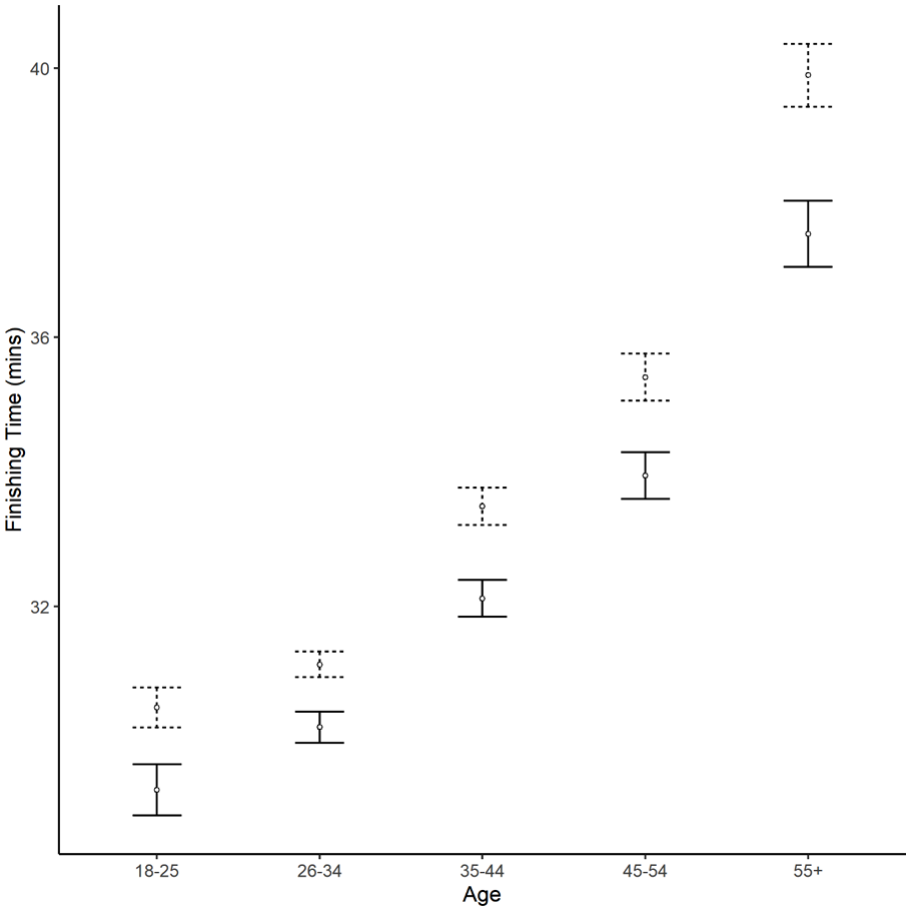
The mean finishing time of new parkrun participants in different age categories before (solid) and after (dashed) the introduction of the PI. Error bars display standard errors.

The most important interaction term was between two social factors, the number of participants and the proportion of new adult participants. At larger events, with higher proportions of new adult participants, finishing times were disproportionately slower. The next interaction term was between remoteness and the number of participants with remote events with larger numbers of participants having disproportionately slower finishers. The next four interaction terms all included age. Older participants were disproportionately slower at more remote events, after the introduction of the PI and at larger events (Figure 3). Women were also disproportionately slower as their age increased than men. The level of adoption of the PI was more strongly associated with slowing down finishing times at larger events. Finally, men had disproportionately slower finishing times at larger events.

### The gender of new parkrun participants in Scotland

The most important factor in the model of gender was the number of participants at an event with an increasing proportion of females at larger events (Table 4). The PI was the second most important factor and was associated with an increase in the proportion of females. Males preferred faster surfaces compared to women. There was also a higher proportion of females at more remote events.

**Table 4.** A GLMM of the proportion of males in adult first-time participants to parkrun in Scotland. All continuous explanatory variables were scaled. Covariates listed in Table 2 but not present in this table were not significantly associated with the proportion of males and were excluded from the final model.

Parameter	*Z*	Estimate	Standard error	*p*
Intercept	4.16	−0.211	0.051	<0.001
Number of participants	4.60	−0.099	0.022	<0.001
Parkwalk	3.39	0.080	0.024	<0.001
Surface	2.46	0.056	0.023	0.014
Remoteness	2.21	0.040	0.018	0.027

### The age of new parkrun participants in Scotland

The age of new participants has been reducing over the 2-year study period. This was best modelled as a consistent decline over time rather than by an interrupted time series associated with the introduction of the PI (parameter estimate = −0.875, s.e. = 0.089, *p* < 0.001). The level of adoption of the PI was associated with a significant increase in age with a slower rate of decline in age associated with level of adoption (parameter estimate = 0.502, s.e. = 0.173, *p* = 0.004).

## Discussion

### Has the PI impacted attendance figures and the number of new participants?

The models reveal that increases in both attendance and the number of new participants are better explained by a change in level at the point the time series was interrupted by the introduction of the PI than by a linear increase over time. This is consistent with a substantial component of the increases in finishing times occurring as a direct result the introduction of the PI. The fact that the level of adoption of the PI by individual events was also positively associated with increases in attendance also provides additional support to the conclusion that the PI has increased attendance rates at parkruns in Scotland.

### The finishing time of new parkrun participants in Scotland

The primary purpose of the PI was to increase the number of walkers at events. The proportion of the participants who walked increased by nearly 50% after the introduction of the PI. Interestingly the proportion of slower runners also increased suggesting that the PI might have also removed barriers to attendance for this cohort.

Several individual, social and physical factors were found to be associated with the finishing time of new participants. Finishing times of new participants at parkrun are likely to be primarily determined by level of fitness and their current level of PA.^41^ The exact contribution of each type of factor to the model is complex to determine because there are many interactions between them, however individual factors can be identified as having the strongest associations with female and older new participants running slower times. This is consistent with other studies of PA.^20^ Both physical factors and social factors also are significantly associated with finishing times. The most strongly associated physical factor was venue with remoter events having slower finishing times. Four social factors were found to be associated with finishing times revealing that events with a higher proportion of new adult participants and larger events had slower finishing times. Discovering multiple important social factors associated with PA is also consistent with a previous study of levels of leisure time PA in Nepal.^17^ The association with event size identified here could simply be related to the logistics of being at a larger event, for example, on average it will take longer to cross the start line at larger events. The association of finishing times with the proportion of other new participants suggests a common sense of camaraderie might play a role in encouraging new participants to feel more comfortable completing parkrun at a slower pace.^58^ The PI is also associated with slower finishing times suggesting that parkrun have successfully manipulated the social environment of their events in the manner intended. This is additionally supported by the fact that the level of adoption of the PI by individual events was associated with the rate of slowing of finishing times. It is also noteworthy that interactions between social factors also explain a considerable proportion of the variation in finishing times. This is encouraging as it suggests that manipulations of the social environment could potentially have multiple impacts through altering the way that existing social factors influence finishing times. The association between finishing times and the remoteness of events suggests that different cultures with respect to finishing times might be able to develop at the more isolated events. The PI also seemed to be more effective at increasing the proportion of female participants at remoter events. This could be related to remoter events having parkrun communities more welcoming to slower participants and female participants, but it could also be related to the wider communities that these events are situated within. More urban events might be drawing more participants from deprived communities with increased barriers to participation, therefore the associations with remoteness could also be explained by differences in the surrounding population and their willingness to participate in PI.

The interaction between age and the PI suggests that it has been more effective at reducing the finishing times of older new participants. A previous study has identified concerns about feeling too unfit to participate are a key barrier to engagement with parkrun.^37^ This suggests that older female potential participants, who might previously have encountered a barrier to engagement in parkrun through a concern about being too slow, might now have completed their first parkrun as a result of the introduction of the PI. The level of adoption of the PI was more strongly associated with slowing down finishing times at larger events suggesting that the PI could be more effective at events with a larger field. One explanation for this is that new participants could have been more easily identified before the PI at smaller events, allowing volunteers and establish participants to engage in them in conversation, creating less of a need for the social engagement component of the parkwalker role at smaller events. Many events that did not adopt the PI in Scotland were smaller events potentially because they already had established ways of engaging with new participants and encouraging walking and therefore did not see an additional benefit from including a parkwalker. It should also be noted that smaller events are also those who are likely to struggle to fill other key volunteer roles. The PI seems to be more effective at larger events where new participants would be harder to identify by providing a new mechanism for established runners and volunteers to engage in conversation with new participants.

The study was restricted to new participants but the increases in attendance at events associated with the introduction of the PI could also include former participants who felt uncomfortable completing parkrun at their pace.^34,37^ The PI could have alleviated their concerns and removed that barrier to participation.

The PI was also associated with an increase in the proportion of new female participants. As women experience increased barriers to participation through feeling too unfit to participate,^37^ they are likely to have particularly benefitted from the introduction of the PI. The fear of being negatively evaluated from being relatively slow might also explain the increased proportion of women at larger events where they might feel less conspicuous and where there is also more chance of other slow participants being present.

Prior to the pandemic the average age at parkrun events in Scotland had been increasing.^33^ This study shows that the average age of new participants has recently been falling. The findings of this study suggest that the PI could have helped slow this fall in age of new participants, potentially by making older, slower, new participants feel more comfortable about taking part in parkrun.

The evidence from this study suggests the PI has been a great success. However, not all events are engaging with it. Smaller events might not gain any benefit from a parkwalker as they might well already be filling that role by being able to identify and engage with new participants. The benefits are seemingly greater at larger events. Those larger events not engaging with the PI could be encouraged to do so. Events adopting the PI might want to consider introducing their parkwalkers at the first timers briefing so new participants are aware of who the parkwalkers are, and what their role entails. The briefing could encourage other walkers to join the parkwalkers. Another strategy might involve advertising “parkwalk at parkrun” to give it a more unique identity. A walking statistic could even be introduced where anyone recording a finishing time of, for example, greater than 50 min gets a walking credit in addition to a participation credit. Alternatively, an opt out of being given a time could be introduced where people choose to opt out via their parkrun profile, so their time is not recorded publicly.

### Strengths and limitations

Many studies of behavioural responses to health interventions have been framed with behavioural models such as social cognitive theory, RE-AIM or socio-ecological models.^28^ These models often include interactions between individual and environmental factors as studied here. Traditional models try to understand environmental influences on behaviour by collecting detailed data about individual contexts and outcomes. This means the quality of the data is high, but the quantity is limited by the number of participants. These studies have often been applied when health initiatives have first been implemented.^25^ The present study adopted a different approach of measuring the response of a very large set of cohorts to a change in the social environment of a long-term existing health intervention. This approach means that the study cannot easily be framed within traditional models due to the lack of data on individual contexts. For example, there is no measure of family level interventions that would be expected in a socio-ecological model and no measure of individual behavioural traits such as self-efficacy that form the basis of social cognitive theory. There are though key overlaps with traditional models as the study investigated individual responses to a change in the social environment of a health intervention. Although the current study lacked the individual context of other studies this allowed this study to be conducted on a huge scale. It measured a population level response for all new parkrun participants in a real-world context for an entire country, Scotland, over a 2-year period in response to a change in a long-term and highly successful health promotion initiative. This makes the study novel, if not, unique. The very large sample size provided high statistical power for identifying associations and interactions, however, another limitation of the study is that it is solely based upon observed correlations within a real-world dataset rather than responses to designed experimental treatments.

Another limitation of the study is that individual parkwalker volunteers might well have varied in how well they engaged with the role. This is not something that would have been recorded at events but it likely to have introduced noise into the dataset and which also would have impacted how well the PI functioned at different events.

## Conclusion

The study has shown that large numbers of individual, physical and social factors interact to determine the finishing time of new parkrun participants. The study clearly shows that interventions to manipulate one aspect of the environment can have profound consequences by not only directly altering engagement with an ALE but also influencing how other factors affect engagement. The PI provides an excellent example of a highly successful intervention that seems to have manipulated engagement with an active leisure event in the way intended. It shows how one barrier to engagement with PA, fear of being negatively evaluated as a result of not being fast enough, can potentially be overcome. This is not just relevant to parkrun and other organisers of active leisure events but also could provide a framework for increasing engagement in PA more broadly by manipulating the social environment of those doing too little PA. Practitioners who engage in social prescription of parkrun might want to prioritise sending patients to parkrun events that adopt the PI. More widely the findings of this study suggest that PA in a group context with encouragement from within the group is more effective than lone PA. Although parkrun is a group activity many walkers do end up walking events on their own as most participants are running. Introducing parkwalkers to engage with other walkers does seem to have successfully enhanced the efficacy of parkrun as a health promotion intervention. Therefore, future initiatives aimed at encouraging walking might be more effectiveness is they promote group walking rather than lone walking.

## References

[1] Grandes G García-Alvarez A Ansorena M , et al Any increment in physical activity reduces mortality risk of physically inactive patients: prospective cohort study in primary care. Br J Gen Pract 2023; 73: e52–e58. 10.3399/BJGP.2022.0118 PMC9639597 36316160

[2] Murray CJL Aravkin AY Zheng P , et al Global burden of 87 risk factors in 204 countries and territories, 1990–2019: a systematic analysis for the Global Burden of Disease Study 2019. Lancet 2020; 396: 1223–1249. 33069327 10.1016/S0140-6736(20)30752-2 PMC7566194

[3] Ramirez Varela A Hallal P Pratt M , et al Global observatory for physical activity (GoPA!): 2nd physical activity almanac. Global Observatory for Physical Activity (GoPA!), 2021. 10.1123/jpah.2024-0233 38621673

[4] Sallis R Young DR Tartof SY , et al Physical inactivity is associated with a higher risk for severe COVID-19 outcomes: a study in 48 440 adult patients. Br J Sports Med 2021; 55: 1099–1105. 33849909 10.1136/bjsports-2021-104080

[5] Pratt M Perez LG Goenka S , et al Can population levels of physical activity be increased? Global evidence and experience. Prog Cardiovasc Dis 2015; 57: 356–367. 25304047 10.1016/j.pcad.2014.09.002 PMC4749397

[6] Bull FC Al-Ansari SS Biddle S , et al World Health Organization 2020 guidelines on physical activity and sedentary behaviour. Br J Sports Med 2020; 54: 1451–1462. 33239350 10.1136/bjsports-2020-102955 PMC7719906

[7] Guthold R Stevens GA Riley LM , et al Worldwide trends in insufficient physical activity from 2001 to 2016: a pooled analysis of 358 population-based surveys with 1·9 million participants. Lancet Glob Health 2018; 6: e1077–e1086. 10.1016/S2214-109X(18)30357-7 30193830

[8] Kohl HW Craig CL Lambert EV , et al The pandemic of physical inactivity: global action for public health. Lancet 2012; 380: 294–305. 22818941 10.1016/S0140-6736(12)60898-8

[9] Bauman AE Reis RS Sallis JF , et al Correlates of physical activity: why are some people physically active and others not? Lancet 2012; 380: 258–271. 22818938 10.1016/S0140-6736(12)60735-1

[10] D’Amore C Bhatnagar N Kirkwood R , et al Determinants of physical activity in older adults: an umbrella review protocol. JBI Evidence Synthesis 2021; 19: 2883–2892. 34074906 10.11124/JBIES-20-00292

[11] Chastin SF Buck C Freiberger E , et al Systematic literature review of determinants of sedentary behaviour in older adults: a DEDIPAC study. Int J Behav Nutr Phys Act 2015; 12: 127. 26437960 10.1186/s12966-015-0292-3 PMC4595239

[12] Alvarez-Lourido D Paniza Prados JL Álvarez-Sousa A. Ageing, leisure time physical activity and health in Europe. Healthcare 2023; 11: 1247. 37174789 10.3390/healthcare11091247 PMC10178047

[13] Fleury J Lee SM. The social ecological model and physical activity in African American women. Am J Community Psychol 2006; 37: 129–140. 16680541 10.1007/s10464-005-9002-7

[14] Lee J Kim Y. A meta-analysis of social ecological correlates of physical activity among Koreans. Percept Mot Skills 2022; 129: 1826–1837. 36112888 10.1177/00315125221126775

[15] Lee Y Park S. Understanding of physical activity in social ecological perspective: application of multilevel model. Front Psychol 2021; 12: 622929. 33746840 10.3389/fpsyg.2021.622929 PMC7973361

[16] Davison RCR Cowan DT . Ageing, sport and physical activity participation in Scotland. Frontiers in Sports and Active Living 2023; 5: 1213924. 37822970 10.3389/fspor.2023.1213924 PMC10562595

[17] Paudel S Owen AJ Smith BJ. Socio-ecological influences of leisure-time physical activity among Nepalese adults: a qualitative study. BMC Public Health 2021; 21: 1443. 34294069 10.1186/s12889-021-11484-3 PMC8296660

[18] Gage R Mizdrak A Richards J , et al The epidemiology of domain-specific physical activity in New Zealand adults: a nationally representative cross-sectional survey. J Phys Act Health 2023; 20: 909–920. 37290767 10.1123/jpah.2022-0156

[19] Heinrich KM Haddock CK Jitnarin N , et al Perceptions of important characteristics of physical activity facilities: implications for engagement in walking, moderate and vigorous physical activity. Front Public Health 2017; 5: 319. 10.3389/fpubh.2017.00319 PMC5712314 29234664

[20] Mauchley O Dinehart C Ahmed S , et al Lifestyles of highly active older adults before and during the COVID-19 pandemic: a qualitative study based on the socio-ecological model. Act Adapt Aging 2023; 0: 1–16.

[21] Haake S Heller B Schneider P , et al The influence of neighbourhood equity on parkrunners in a British city. Health Promot Int 2022; 37: daab138. 10.1093/heapro/daab138 34486666

[22] Loureiro N Calmeiro L Marques A , et al The role of Blue and green exercise in planetary health and well-being. Sustainability 2021; 13: 10829.

[23] Anderson J Benton JS Ye J , et al Large walking and wellbeing behaviour benefits of co-designed sustainable park improvements: a natural experimental study in a UK deprived urban area. Environ Int 2024; 187: 108669. 38677084 10.1016/j.envint.2024.108669

[24] Veitch J Ball K Crawford D , et al Park improvements and park activity: a natural experiment. Am J Prev Med 2012; 42: 616–619. 22608379 10.1016/j.amepre.2012.02.015

[25] Baba CT Oliveira IM Silva AEF , et al Evaluating the impact of a walking program in a disadvantaged area: using the RE-AIM framework by mixed methods. BMC Public Health 2017; 17: 1–11. 28915827 10.1186/s12889-017-4698-5 PMC5603090

[26] Richards EA Woodcox S. A county extension-delivered, email-mediated walking intervention: a programme evaluation. Health Educ J 2018; 77: 615–624.

[27] Frensham LJ Parfitt G Dollman J. Predicting engagement with online walking promotion among Metropolitan and Rural Cancer Survivors. Cancer Nurs 2020; 43: 52–59. 30312190 10.1097/NCC.0000000000000649

[28] Cleland CL Tully MA Kee F , et al The effectiveness of physical activity interventions in socio-economically disadvantaged communities: a systematic review. Prev Med 2012; 54: 371–380. 22521997 10.1016/j.ypmed.2012.04.004

[29] Hillman P Lamont M Scherrer P , et al Reframing mass participation events as active leisure: implications for tourism and leisure research. Tour Manag Perspect 2021; 39: 100865.

[30] Hindley D. Parkrun: an organised running revolution. Routledge & CRC Press, 2022, https://www.routledge.com/parkrun-An-Organised-Running-Revolution/Hindley/p/book/9780367640613 (accessed 5 April 2023).

[31] Haake S Quirk H Bullas A. Parkrun and the promotion of physical activity: insights for primary care clinicians from an online survey. Br J Gen Pract 2022; 72: e634–e640. 10.3399/BJGP.2022.0001 PMC9423046 35995575

[32] Wingate S Sng E Loprinzi PD. The influence of common method bias on the relationship of the socio-ecological model in predicting physical activity behavior. Health Promot Perspect 2018; 8: 41–45. 29423361 10.15171/hpp.2018.05 PMC5797307

[33] Gilburn AS. New parkrunners are slower and the attendance gender gap narrowing making parkrun more inclusive. Int J Environ Res Public Health 2023; 20: 3602. 36834295 10.3390/ijerph20043602 PMC9959326

[34] Gilburn AS. Predictors of successful return to parkrun for first-time adult participants in Scotland. PLOS Global Public Health 2023; 3: e0001786. 10.1371/journal.pgph.0001786 PMC10431652 37585404

[35] Grunseit AC Huang B-H Merom D , et al Patterns and correlates of participation in a weekly mass participation physical activity event, parkrun, in Australia, 2011–2020. J Phys Act Health 2024; 21: 155–163. 38134894 10.1123/jpah.2023-0532

[36] Malchrowicz-Mośko E León-Guereño P Tapia-Serrano MA , et al What encourages physically inactive people to start running? An analysis of motivations to participate in Parkrun and City Trail in Poland. Front Public Health 2020; 8: 581017. 33313036 10.3389/fpubh.2020.581017 PMC7707109

[37] Reece LJ Owen K Graney M , et al Barriers to initiating and maintaining participation in parkrun. BMC Public Health 2022; 22: 83. 35027014 10.1186/s12889-022-12546-w PMC8759213

[38] Cleland V Nash M Sharman MJ , et al Exploring the health-promoting potential of the “parkrun” phenomenon: what factors are associated with higher levels of participation? Am J Health Promot 2019; 33: 13–23. 29685052 10.1177/0890117118770106

[39] Fullagar S Petris S Sargent J , et al Action research with parkrun UK volunteer organizers to develop inclusive strategies. Health Promot Int 2020; 35: 1199–1209. 31778185 10.1093/heapro/daz113

[40] Smith RA Schneider PP Cosulich R , et al Socioeconomic inequalities in distance to and participation in a community-based running and walking activity: a longitudinal ecological study of parkrun 2010 to 2019. Health Place 2021; 71: 102626. 34333371 10.1016/j.healthplace.2021.102626 PMC8522482

[41] Stevinson C Hickson M. Changes in physical activity, weight and wellbeing outcomes among attendees of a weekly mass participation event: a prospective 12-month study. J Public Health 2019; 41: 807–814. 10.1093/pubmed/fdy178 30295838

[42] Mulvenna M Adie JW Tramontano C. Self-based goals, underlying reasons, performance and discrete emotions among parkrunners. Front Psychol 2023; 14: 1017836. 37465486 10.3389/fpsyg.2023.1017836 PMC10352087

[43] Davis AJ MacCarron P Cohen E. Social reward and support effects on exercise experiences and performance: evidence from parkrun. PLoS One 2021; 16: e0256546. 10.1371/journal.pone.0256546 PMC8443045 34525097

[44] Hindley D. “More than just a run in the park”: an exploration of parkrun as a shared leisure space. Leisure Sci 2020; 42: 85–105.

[45] Stevinson C Wiltshire G Hickson M. Facilitating participation in health-enhancing physical activity: a qualitative study of parkrun. Int J Behav Med 2015; 22: 170–177. 25096794 10.1007/s12529-014-9431-5

[46] Wiltshire GR Fullagar S Stevinson C. Exploring parkrun as a social context for collective health practices: running with and against the moral imperatives of health responsibilisation. Sociol Health Illn 2018; 40: 3–17. 28990198 10.1111/1467-9566.12622

[47] Stevens M Rees T Polman R. Social identification, exercise participation, and positive exercise experiences: evidence from parkrun. J Sports Sci 2019; 37: 221–228. 29912669 10.1080/02640414.2018.1489360

[48] Warhurst R Black K. Lost and found: parkrun, work and identity. Qual Res Sport Exerc Health 2022; 14: 397–412.

[49] Wiltshire G Stevinson C. Exploring the role of social capital in community-based physical activity: qualitative insights fromparkrun. Qual Res Sport Exerc Health 2018; 10: 47–62.

[50] Gilburn A. Green exercise, blue spaces and active leisure events: the performance of new participants is associated with their response to event characteristics. J Glob Sport Manag 2024; 10: 514–533.

[51] Gilburn AS. Exercise should be blue and green: seasonal variation in how woodland and freshwater interact to enhance participation in active leisure events. Urban For Urban Green 2025; 112: 128917.

[52] Quirk H Haake S. How can we get more people with long-term health conditions involved in parkrun? A qualitative study evaluating parkrun’s PROVE project. BMC Sports Sci Med Rehabil 2019; 11: 22. 31636909 10.1186/s13102-019-0136-6 PMC6798471

[53] parkrun UK Blog. Parkwalker role explained, https://blog.parkrun.com/uk/2022/09/26/parkwalker-role-explained/ (2022, accessed 2 May 2023).

[54] The R Project. R: The R Project for Statistical Computing, https://www.r-project.org/ (2023, accessed 12 April 2023).

[55] Bates D Mächler M Bolker B , et al Fitting linear mixed-effects models using LME4. J Stat Softw 2015; 67: 1–48.

[56] Marquardt DW. Comment: you should standardize the predictor variables in your regression models. J Am Stat Assoc 1980; 75: 87–91.

[57] Vu DH Muttaqi KM Agalgaonkar AP. A variance inflation factor and backward elimination based robust regression model for forecasting monthly electricity demand using climatic variables. Appl Energy 2015; 140: 385–394.

[58] Walsh DW Green BC Harrison T , et al ‘Sport as a resource caravan’: understanding how adults utilize sport as a developmental tool. J Glob Sport Manag 2022; 7: 546–568.

